# The Art of Living with NZT and ICT: Dialectics of an Artistic Case Study

**DOI:** 10.1007/s10699-015-9438-7

**Published:** 2015-10-17

**Authors:** Hub Zwart

**Affiliations:** P.O. Box 9010, 6500 GL Nijmegen, Netherlands

**Keywords:** Dialectics, Philosophy of technology, Brain cinema, Genres of the imagination, Neuro-enhancement

## Abstract

I wholeheartedly sympathize conceptually with Coeckelbergh’s paper. The dialectical relationship between vulnerability and technology constitutes the core of Hegel’s Master and Slave (the primal scene of contemporary philosophy). Yet, the empirical dimension is underdeveloped and Coeckelbergh’s ideas could profit from exposure to case studies. Building on a movie/novel (*Limitless*) devoted to vulnerability coping and living with ICT, I challenge the claim that modern heroism entails overcoming vulnerability with the help of enhancement and computers.

Mark Coeckelbergh convincingly regards the ethics and aesthetics of living with ICT as “vulnerability coping”: a matter of both ethics and art, both morality and style. Living-with-ICT is seen as a contemporary way of being-in-the-world, so that human existence fundamentally means *being*-*at*-*risk*. New technologies will *transform*, but never *erase* human vulnerability, although various strategies of “vulnerability coping” may be subjected to a critical comparative analysis. Special attention is given to the human enhancement-debate. Transhumanism is seen as an “anti-vulnerability program”, a product of modern culture as an “anti-vulnerability culture”.

For me, as a ‘continental’ philosopher of technoscience, this line of reasoning is quite congenial. In fact, existential vulnerability is already the starting-point of Hegel’s phenomenological-dialectical narrative of Master and Slave (which I still regard as the ‘primal scene’ of contemporary philosophy). The Master is vulnerable from the very outset, unable himself to produce and process the items needed for his own subsistence (Hegel 1807/1970/1986). As a gesture of despair, he throws himself into a dramatic decisive struggle, putting his life at risk, and initially, this seems to work well. Temporarily at least, he seems able to rely on the work of the Slave (who is initially casted as the loser), on the tools and technologies the latter develops/deploys. Whereas the Master is allegedly autonomous, the Slave is supposedly heteronomous (subjected to the Master’s will). Gradually, however, due to the tools and technologies he develops and puts to use, the Slave becomes increasingly powerful and autonomous, and the Master increasingly vulnerable and dependent.

We may read this as a simile for the human-technology relationship as such. Initially, technologies (such as ICTs) are seen as instrumental (acquired and ‘used’ by us, for our own benefit). We decide to use them even if that means accepting the struggles and risks involved. Gradually, we become increasingly dependent on the technologies which enable a particular way of being-in-the-world. They increasingly manage to re-sculpt our bodies and lives. While natural voices and ears, for instance, only allow us to communicate within the here and now, ICT opens up a much broader realm, allowing us to communicate far beyond natural restrictions of space and time, enabling us to become who we are as hypermodern individuals, enabling us to function in a global environment. Yet, once addicted to ICT, new forms of vulnerability and risk are bound to proliferate.

In short, I am basically in agreement with this paper (a piece of “beautiful craftsmanship”). Its basic ‘poverty’ for me resides in the ‘empirical’ dimension. Inspiring ideas are developed on a fairly general level. They are not really tested/elaborated via systematic exposure to case studies. This is what I purport to do in my brief commentary: expose the author’s arguments to a concrete artistic case of living-with-ICT. In view of the convergence of the moral and the aesthetical dimension, ‘genres of the imagination’ (i.e. novels, movies, drama and the like) provide ample case material for such an exercise. I have selected one recent movie/novel for this purpose, which not only addresses (cognitive) enhancement, but also the various forms of living-with-ITC associated with it.

By doing so, it will become clear that, as soon as the empirical dimension is taken more seriously, the world is less straightforward than the (quietly theorizing) author presumes. In fact, at the point where his paper does become relatively concrete, I tend to disagree with him, namely where he speaks about modern cinematic heroes. According to the author, the typical modern hero “is very powerful and has limited vulnerability, limited emotional involvement, and limited ties to others (think about protagonists in contemporary action films)”. Building on a recent action movie, featuring a typical modern hero struggling in a world of ICT, I will argue that this is not the case. Quite the contrary, ‘vulnerability’ rather than ‘power’ remains the quintessence of hypermodern subjectivity, even in techno-thrillers. The focus, I will argue, is on the frantic (but inevitably faltering) efforts to cope with inherent, unsurmountable human vulnerability, notably in the face of ICT-related risks.

As a case study, I have chosen the movie *Limitless*, based on the novel *The Dark Fields* by Glynn (republished as *Limitless* in 2011), telling the story of a middle-aged writer named Eddie Morra who, having finally secured a book-contract with a Manhattan publisher, falls victim to a writers’ block (Glynn 2001/2011). A nootropic wonder-drug named NZT allows him to lift his inhibitions, so that he not only finishes his book, but also becomes a stock market virtuoso. Although *Limitless* is primarily about brain enhancement, it addresses ICT as well. Or, rather, it unravels the various ways in which neuro-enhancement and ICT are intimately and mutually interconnected.

From the very outset, Eddie desperately tries to cope with human vulnerability. At the beginning of the movie, he struggles with language, with the first sentence of his unwritten novel: an alcohol-dependent artist in front on his laptop, unable to produce anything, while elsewhere in his room the telephone relentlessly sends out frightening signals (his publisher, reminding him of expiring deadlines). In other words, ICT items meant to facilitate his productivity (laptop, telephone) become sources of anxiety. Eddie is unable to live up to the personal ambitions and societal expectations mediated through these contrivances.

Suddenly, something unexpected happens. A former acquaintance offers him a wonder-drug, allowing him to mobilise his faltering creativity and to streamline his deficient brain. The drug completely alters his way of being-in-the-world, the tonality of his existence. It enables him to interact much more effectively with ICT. His NZT-soaked brain increasingly mimics and merges with computers. He becomes so good with computers that he decides to play the stock-market. Due to his ability to process huge amounts of information, he becomes the new hero of the trading scene (p. 122). Gradually, his electronic ruminations coalesce into “an overwhelming vision of the vastness and beauty of the stock-market itself”. He sees it as “a celestial firmament”. With its “complexities and ceaseless motion, the 24-h global network of trading systems was nothing less than a template for human consciousness … a collective nervous system, a global brain” (p. 123). Due to the sudden smoothness of the collaboration between machine and man, microchips and cells, circuits and synapses, he realizes “a grand convergence of band-width and brain-tissue”, giving rise to the idea that his mind works “like a living fractal” (p. 123). Eventually, he becomes adviser and partner of the most powerful man of all Manhattan: venture capitalist Carl van Loon. Eddie masterminds the merger of two very large Manhattan firms: a process of staggering complexity, beyond the grasp of ordinary individuals.

So far, the movie/novel seems in complete agreement with Coeckelbergh’s verdict that modern heroes, notably in contemporary action films, are very powerful and have limited vulnerability. Yet, on closer inspection, Eddie’s experience of invulnerability and power proves transitory and illusory. The risks involved in the living-with-ICT which he enacts are *transformed*, significantly *amplified* even, rather than erased or reduced. Very soon, his chronic and insurmountable vulnerability is painfully revealed. For a short period of time, Eddie occupies the position of the Master, accepting the life-threatening risks involved (addiction to an enigmatic neuro-pharmaceutical, and its increasingly detrimental side-effects, but also the hazards involved in the world of high finance, such as blackmailing criminals). But in the end, his provision of wonder-pills proves finite.

Eddie’s vicissitudes can be described in a topological manner. Initially, he seems completely decentralised: a nameless writing atom adrift in Manhattan. At a certain point, he moves towards the centre of the modern financial ICT-dense world. But his performance within this global network is highly dependent on a combination of biopharmaceutical and information and communication technologies (Zwart 2014).

Interestingly, the novel and the movie opt for different endings. In the novel, Eddie dies a miserable death as a fugitive suffering from paralyzing withdrawal symptoms, when the pill supply is discontinued for good. In the movie, he seems about to run for President. But would that change the picture? Not really. It is not a coincidence, I guess, that the current US President is a President without a Congress, disconnected from ‘the system’, governing via back-up systems (decrees), still attempting to live with vulnerability.
